# Development and validation of a comprehensive complication index for predicting outcomes in fournier’s gangrene

**DOI:** 10.1007/s11845-026-04405-z

**Published:** 2026-04-29

**Authors:** Fatih Gokalp, Eser Ordek, Muhanned Sammala, Duran Kuru, Ferhat Ucurmak, Sadik Gorur

**Affiliations:** Department of Urology, Faculty of Medicine, Hatay Mustafa Kemal University, Antakya, Hatay 31100 Türkiye

**Keywords:** Fournier gangrene, Complication, Comprehensive complication index

## Abstract

**Backgrounds:**

Fournier’s gangrene is a life-threatening condition. To predict patient outcomes, it requires a thorough understanding of the variables influencing the severity of this illness and the emergence of comorbidities.

**Aims:**

To evaluate the Comprehensive complication index score to predict postoperative mortality in Fournier gangrene.

**Methods:**

Between 2013 and 2024, a retrospective analysis was used to identify patients who were diagnosed with Fournier’s gangrene. The perioperative and clinical data were recorded. Postoperative complications were assessed using the Clavien-Dindo classification (CDC). The Comprehensive Complication index was calculated based on CDC and when multiple complications occurred, their values were combined using the CCI formula to generate a cumulative score for each patient. Upgrading was interpreted as a CCI cumulative score surpasses the baseline score. The data was divided into two groups based on patients’ mortality outcome; group 1: survive group, group 2: mortality group

**Results:**

The age and comorbidities were significantly higher in group 2, (p<0.001 and p=0.015). The frequency of sepsis was considerably higher in group 2 patients (p<0.001). The CCI score significantly higher in group 2 (p<0.001). ROC analysis showed that the CCI score greater than 36.60 could accurately predict mortality with high specificity and sensitivity (91.3% and 70.0%, respectively). Multivariate analysis identified both fage (OR: 1.436, p = 0.002) and CCI score (OR: 1.093, p = 0.008) as independent predictors of mortality.

**Conclusion:**

The CCI is a useful instrument for evaluating cumulative postoperative morbidity and forecasting mortality in patients with Fournier’s gangrene. Incorporating the CCI into clinical practice may enhance risk classification, optimize resource allocation, and inform treatment decision-making in FG management.

## Introduction

Fournier’s gangrene (FG) is a potentially life-threating that is characterized by a fast progression of necrotizing fasciitis that primarily affects the genital areas [1]. FG generally arises from a genital infection that can expand rapidly and evolve into severe sepsis and multiorgan failure [2]. Despite advances in critical care, broad-spectrum antibiotics, and surgical management, FG continues to be associated with high morbidity and a global mortality rate reported between 20% and 40%, underscoring the importance of early risk stratification and effective clinical decision-making [1, 2]. Improving patient outcomes requires a thorough understanding of the variables influencing the severity of this illness and the emergence of comorbidities. The Clavien-Dindo classification (CDC) is commonly used to classify postoperative surgical complications [3]. However, instead of taking into consideration the cumulative risk of complications, the CDC does not completely count complications that patients faced multiple complications. The Comprehensive Complication Index (CCI) was created to mainly overcome this restriction [4]. Existing literature has primarily focused on the CCI in relation to various cancer outcomes, demonstrating that the CCI is an effective predictor of both mortality and morbidity in urogenital and gastrointestinal cancers and also provides postoperative evaluation of surgical techniques [5–8].

Identifying individuals at increased risk for negative outcomes, the creation of a thorough complication index may offer important insights into the clinical progression of Fournier’s gangrene. Especially, predicting clinical severity and mortality are still crucial for Fournier gangrene [9]. Some studies describe a scoring systems to predict mortality [10, 11]. Fournier gangrene severity index (FGSI) FGSI, which is calculated from clinical and laboratory parameters, has nine grading scores for each parameter [11, 12]. FGSI was defined as a predictor of mortality. However, FGSI ignores important factors such as the patient’s age or dissemination of the disease [10]. Although these scores targeted at predicting mortality by assessing the severity of the disease, existing or occurring complications were ignored. Since complications themselves are major contributors to poor outcomes, especially mortality, a scoring system that captures their total burden may provide clinically valuable insight.

Given this gap in the literature, evaluating the usefulness of the CCI in FG may help identify high-risk patients, guide treatment strategies, and improve postoperative monitoring. In this study we tried to demonstrate and evaluate the efficacy of CCI in patients with Fournier gangrene.

## Methods

### Study design and patients’ selection

Between 2013 and 2024, a retrospective cohort analysis was done on patients diagnosed Fournier’s gangrene at university hospital. Approval for the study was received from the local ethics committee numbered [2024/#35] (Hatay Mustafa Kemal University Clinical Research Ethics Committee). The study was conducted in accordance with the principles of the Declaration of Helsinki, and all patient data were anonymized. Because this was a retrospective study based on electronic medical records, blinding of data collectors to patient outcomes was not done. However, data extraction followed a standardized protocol to minimize bias.

Patients diagnosed with Fournier’s gangrene who underwent surgical debridement between the specified dates were included in the study.

Inclusion criteria: Age 18 years or older and diagnosed with Fournier’s gangrene and undergone surgical debridement were included the study.

Exclusion criteria: Patients under 18 years of age (pediatric) or a history of active cancer or those receiving ongoing chemotherapy/radiotherapy. Patients with more than 20% missing data to assess primary and secondary outcomes were excluded from study.

A systematic procedure is used at our facility for surgical debridement of Fournier’s gangrene. Under general anesthesia, all necrotic, diseased, and non-viable tissues are meticulously removed until healthy bleeding margins are evident. Wound irrigation with saline and antiseptic solution is conducted, and additional debridement sessions are arranged if continued necrotic advancement is observed. The operating surgeon determined the necessity for repeat debridement based on clinical evaluations and wound assessment results. Patients were transferred to the ICU if they presented with sepsis or Hemodynamic instability requiring vasopressor support. Sepsis was defined by the Sepsis-3 criteria as “life-threatening organ dysfunction resulting from a dysregulated host response to infection,” shown by an immediate increase of ≥ 2 points in the SOFA score. In cases where patients did not have complete SOFA data in the retrospective records, a clinical diagnosis of sepsis, as documented by the treating physicians (e.g., hemodynamic instability, septic shock necessitating vasopressors), was deemed acceptable for analysis.

## Data collection and variables

Patients’ demographic, clinical, and perioperative data were retrospectively obtained from the hospital registration system (or electronic patient records). Age, gender, body mass index (BMI, kg/m²), and comorbidities (presence of diabetes mellitus, hypertension, coronary artery disease, etc.) were recorded. Perioperative and clinical data including total number of surgical debridement, length of intensive care unit (ICU) stay, total hospital stay, and whether a colostomy or orchiectomy was performed were recorded. Additionally, patients’ hematological and biochemical parameters at admission (specifically, those required for the FGSI calculation: temperature, heart rate, respiratory rate, serum sodium, potassium, creatinine, bicarbonate, hematocrit, and leukocyte count) were recorded.

Postoperative complications were assessed using the Clavien-Dindo classification (CDC). Each complication was assigned a grade based on severity, ranging from grade I to grade V (death). Mortality was determined as the count of fatalities occurring within 90 days following the initial surgery. The data was divided into two groups based on survivors and non-survivors. Group 1 contained survivors, while Group 2 consisted of non-survivors.

The Comprehensive Complication Index (CCI) calculator, accessible at https://www.cci-calculator.com, was used to determine the CCI. The CCI facilitates a more thorough evaluation of surgical outcomes by providing a cumulative score that combines all of a patient’s problems into a single value. Based on CDC assessment, the following values were allocated to individual grades: Grade I: 8.7; Grade II: 20.9; Grade IIIa: 26.2; Grade IIIb: 33.7; Grade IVa: 42.4; Grade IVb: 46.2; Grade V (death): 100. When multiple complications occurred, their values were combined using the CCI formula to generate a cumulative score for each patient. Increasing in cumulative CCI scores from baseline score were interpreted as an upgrading CCI. The primary outcome of the study was the efficacy of CCI in predicting mortality associated with Fournier’s gangrene. The second outcome of the study was to determine the other predictive factors that can predict mortality in Fournier’s gangrene. The FGSI is a validated scoring system utilized to evaluate the severity and forecast the prognosis of Fournier’s gangrene. This score is derived from deviations in nine physiological and laboratory parameters: temperature, heart rate, respiratory rate, serum sodium, potassium, creatinine, bicarbonate, hematocrit, and leukocyte count. Each variable is assigned a score ranging from 0 to 4 based on the extent of derangement [12].

The study was approved by local ethic committee. All information was anonymized to protect patient privacy.

### Statistical analysis

All statistical analyses were performed using SPSS version 30 for MacOS (IBM Corp., Armonk, NY, USA). Continuous variables were evaluated for normal distribution using the Shapiro-Wilk test. Non-normally distributed continuous variables are presented as median and interquartile range (IQR), while categorical variables are presented as number (n) and percentage (%). For comparisons between Group 1 (Survivors) and Group 2 (Mortality), based on the primary outcome (mortality). The Mann-Whitney U test was used for non-normally distributed continuous variables (e.g., age, CCI, FGSI). For categorical variables (e.g., gender, presence of comorbidities, sepsis), the Chi-square test was used, or, Fisher’s exact test was used.

Multivariate logistic regression analysis was used to identify independent predictors (independent variables) associated with mortality (dependent variable). Variables found to be statistically significant in univariate analysis (e.g., age, presence of comorbidities, sepsis) and baseline scores known to be clinically important (FGSI, CCI) were included in the logistic regression model. Receiver Operating Characteristic (ROC) curve analysis was performed to evaluate the predictive performance of the CCI and FGSI scores for 90-day mortality. The discriminatory power of the models was assessed by comparing the Area Under the Curve (AUC) values. Optimal CCI and FGSI cut-off values ​​for predicting mortality were determined using the Youden Index. Statistical significance was set at *p* < 0.05 for all analyses.

## Results

The median age of the patients was 56 years (IQR = 53–59). Comorbidities were identified in 65.1% of the cohort, with diabetes mellitus being the most prevalent condition. A median of two debridement procedures were performed per patient (IQR = 1–2), and the observed mortality rate was 33.3% (Table 1).

**Table 1:** Demographic data

		Value
Age ^a^		56.00 (53.00–59.00)
BMI ^a^		27.00 (26.00–28.00)
Comorbid disease count ^b^	**Absent**	22 (34.9%)
	**Single**	32 (50.8%)
	**Multiple**	9 (14.3%)
Comorbid diseases ^b^	**Absent**	21 (33.3%)
	**DM**	39 (61.9%)
	**HT**	2 (3.2%)
	**CAD**	0 (0.0%)
	**CKD**	1 (1.6%)
Debridement count ^a^		2 (1–2)
Sepsis ^b^	**Absent**	43 (68.3%)
	**Present**	20 (31.7%)
Colostomy ^b^	**Absent**	43 (70.5%)
	**Present**	18 (29.5%)
Orchidectomy ^b^	**Absent**	55 (90.2%)
	**Present**	6 (9.8%)
Mortality ^b^	**Alive**	40 (63.5%)
	**Death**	23 (36.5%)

^**a**^The variables expressed as median and IQR (interquartile range)

^**b**^The variables expressed as count and frequency

*BMI Body mass index*,* DM Diabetes Mellitus*,* HT hypertention*,* CAD Coronary artery disease*,* CKD Chronic kidney disease.*

There was a statistically significant age difference between the two groups, with group 2 patients being older (*p* < 0.001). Additionally, individuals in group 2 exhibited significantly higher burden of comorbid conditions compared to group 1 (*p* = 0.015) (Table 2). The frequency of sepsis was considerably higher in group 2 patients (*p* < 0.001). However, there was no significant difference in the rates of colostomy (*p* = 0.284) or orchiectomy (*p* = 0.117) between the groups. The median hospitalization date was similar between groups (*p* = 0.070). The Clavien complications were also different between groups. The distribution of postoperative complications differed significantly between groups. Minor complications (Clavien < IIIa): more frequent in survivors (62.3% vs. 21.1%, *p* < 0.001). However, major complications (Clavien ≥ IIIb): more frequent in non-survivors (57.9% vs. 9.4%; *p* < 0.001). The Comprehensive Complication Index (CCI) score was significantly higher in non-survivor group (survivor:39.70 (27.20–47.60) vs. non-survivor: 60.90 (47.60–60.90), *p* < 0.001). Additionally, FGSI score was significantly higher in non-survivor group (survivor: 2.00 (0.50–4.00.50.00) vs. non-survivor: 7.00 (5.00–10.00), *p* < 0.001) and upgrading of the CCI score was significantly more frequent in non-survivor group (*p* = 0.004).

**Table 2:** Comparison of two groups

		**Survivors** **(n=40)**	**Non-survivors** **(n=23)**	**p value**
Age (year) ^a^		54.00 (53.00-57.00)	59.00 (58.00-62.00)	**<0.001&**
BMI ^a>^		27.00 (26.00-28.00)	28.00 (26.00-28.00)	0.054
CRP ^a^		199.50 (190.00-238.0)	177.00 (156.00-211.00)	**0.037&**
Comorbid disease count ^b^	**Absent**	14 (35.5%)	8 (34.8%)	**0.015#**
	**Single**	24 (60.0%)	8 (34.8%)	
	**Multiple**	2 (5.0%)	7 (30.4%)	
Comorbid diseases ^b^	**Absent**	13 (32.5%)	8 (34.8%)	0.236
	**DM**	26 (65.0%)	13 (56.5%)	
	**HT**	0 (8.7%)	2 (8.7%)	
	**CAD**	0 (0.0%)	0 (0.0%)	
	**CKD**	1 (2.5%)	0 (0.0%)	
Debridement count ^a^		2.00 (1.00-2.00)	1.00 (1.00-2.00)	0.540
Orchidectomy ^b^	**Absent**	37 (92.5%)	18 (81.8%)	0.117
	**Present**	3 (7.5%)	4 (18.2%)	
Colostomy ^b^	**Absent**	26 (65.0%)	17 (77.3%)	0.284
	**Present**	14 (35.0%)	5 (22.7%)	
Sepsis ^b^	**Absent**	37 (92.5%)	6 (26.1%)	<0.001*
	**Present **	3 (7.5%)	17 (73.9%)	
Hospitalization date (day) ^a^		21.00 (14.00-28.00)	25.00 (20.00-30.00)	0.070
Clavien complication				**< 0.001#**
	**Clavien 2**	10 (25.0%)	2 (8.7%)	
	**Clavien 3a**	7 (14.5%)	0 (0.0%)	
	**Clavien 3b**	18 (45.0%)	7 (30.4%)	
	**Clavien 4a**	5 (21.5%)	5 (21.7%)	
	**Clavien 4b**	0 (0.0%)	9 (39.1%)	
FGSI		2.00 (0.50-4.00)	7.00 (5.00-10.00)	**<0.001&**
CCI score ^a^		39.70 (27.20-47.60)	60.90 (47.60-60.90)	**<0.001&**
CCI cumulative score upgraded	**Absent**	22 (55.0%)	4 (17.4%)	0.004*
	**Present**	18 (45.0%)	19 (82.6%)	

^**a**^The variables expressed as median and IQR (interquartile range). ^**b**^The variables expressed as count and frequency. Bold values are statistically significant.* Fisher’s exact test was used.# Pearson chi-square test was used.& Mann Whitney U test was used. *BMI: Body mass index, CRP: C-reaktive protein, DM: Diabetes Mellitus, HT: hypertention, CAD: Coronary artery disease, CKD: Chronic kidney disease, CCI: Comprehensive complication index. FGSI: Fournier gangrene severity index.*

**Fig. 1:**
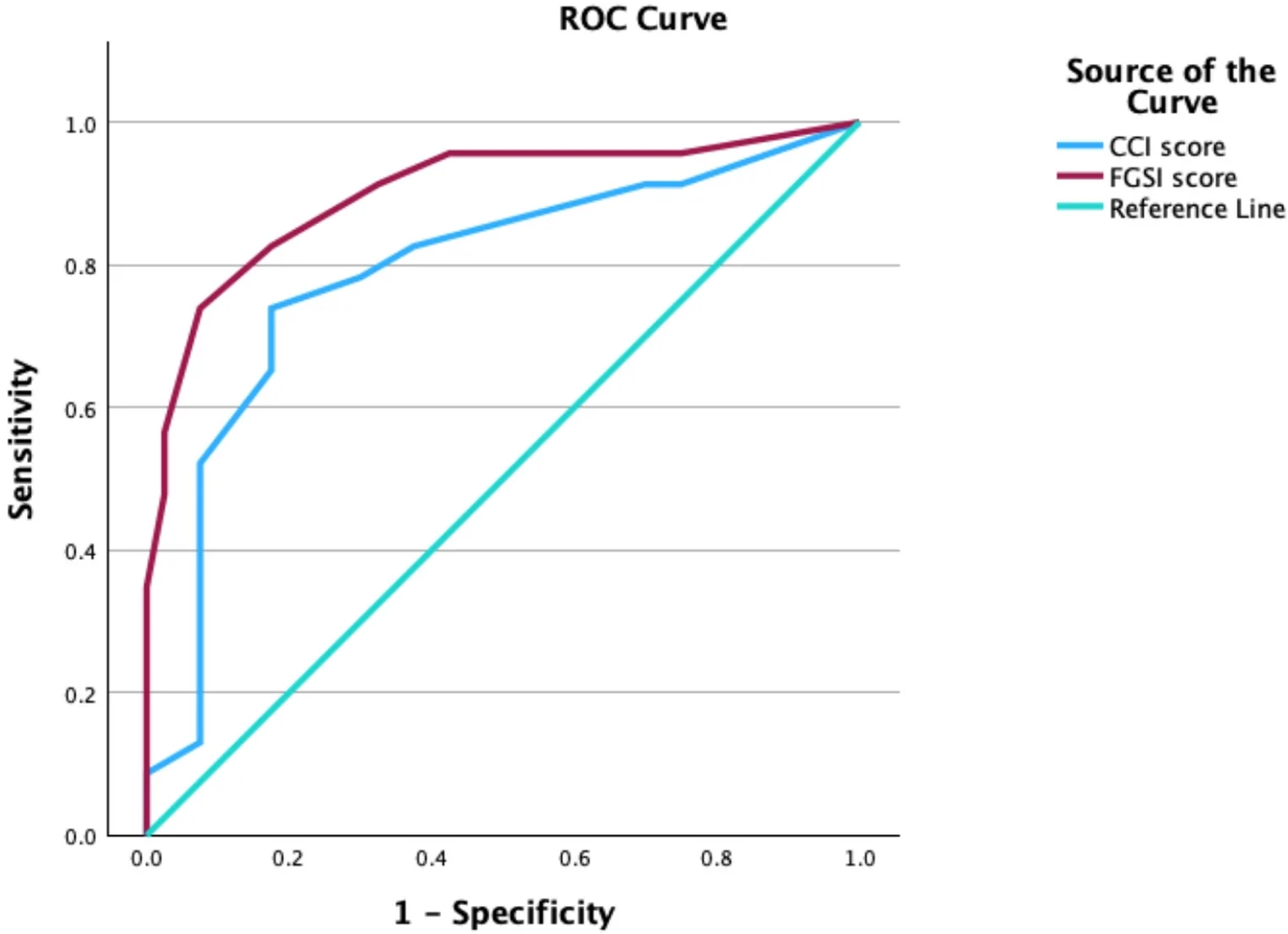
ROC analysis of FGSI and CCI score on mortality

The multivariate analysis showed that FGSI, CCI and age were independent factors for mortality in FG (O.R. 2.098, 95% CI. 1.317–3.345; *p* = 0.002 and O.R. 1.084, 95% CI. 1.001–1.173; *p* = 0.048, and O.R. 1.597, 95% CI. 1.115–2.286; *p* = 0.011, respectively) (Table 3). Additionally, ROC analysis showed that a CCI score greater than 36.60 could accurately predict mortality with high specificity and sensitivity (%91.3 and %70.0, respectively). However, ROC analysis showed that FGSI score was superior factor for predicting mortality when compared CCI score (AUC:0.901, 0.813–0.988 vs. AUC: 0.790, 0.665–0.914, respectively) (Fig. 1).

**Table 3:** Multivariate analysis factors for mortality

**Variables in the Equation**				
		**OR**	**95% C.I. Lower- Upper**	**p value**
Step 1^a^	**FGSI core**	2.098	(1.317 - 3.345)	**0.002**
	**CCI score**	1.084	(1.001- 1.173)	**0.048**
	**Age**	1.597	(1.115 - 2.286)	**0.011**
	**Comorbid disease**			0.260
	**Comorbid disease (1)**	1.730	(0.164 −18.244)	0.648
	**Comorbid disease (2)**	13.587	(0.532 - 347.008)	0.115
	**Constant**	.000		0.004

a. Variable(s) entered on step 1: FGSI, CCI, Age, Comorbid disease.

## Discussion

The Comprehensive Complication Index (CCI) is a useful and simple system for assessing complications following surgery. Especially, in the disease with multiple comorbidities such as Fournier gangrene, the CCI score could predict mortality and morbidity by calculation complication not only categorical. The primary finding of this study is that the Fournier’s Gangrene Severity Index (FGSI), which reflects the patient’s systemic physiology at admission, remains superior predictor of mortality in patients with Fournier’s gangrene (FG) than the CCI. In contrast, the CCI reflects the cumulative burden of postoperative complications. Although the FGSI is a stronger predictor, our study confirmed that CCI and advanced age are also independent risk factors for mortality. This highlights the necessity considering both the patient’s initial physiologic status and the course of postoperative complications in the management of FG.

While significant reductions in mortality rates have been achieved for many diseases, accurate mortality prediction still remains essential, particularly for conditions like FG with inherent risks [13]. The CCI offers a more accurate approach to assessing surgical complexity and risk, providing valuable insights that traditional classification systems may overlook. A lengthy, rigorous, and progressive iterative process led to the creation of the CCI [4]. In contrast to the CCI, the Clavien-Dindo Classification (CDC) relies on an ordinal scale that may oversimplify information about the overall patient morbidity burden and conceals graded numerical assumptions. Furthermore, the majority of studies only report the highest-grade CDC complication, which results in data loss and an underestimation of the overall number of complications for patients with both high- and low-grade complications [14]. The literature also showed CCI could be useful and valuable metric for assessing postoperative complication after urological surgeries [7, 14, 15]. Nevertheless, controversial findings in the literature indicated that CCI was not significantly superior to CDC [15, 16]. Previous studies have demonstrated that CCI provides a comprehensive assessment of complications across various surgical domains. A study by Huang et al. that evaluated 280 elderly patients who had radical cystectomy revealed that CCI, which accounts for all complications, predicted postoperative stay more accurately than the CDC, which only evaluated the most serious complications (*r* = 0.378, *p* < 0.001 vs. *r* = 0.349, *p* < 0.001) [17]. Another novel paper assessed 287 patients who underwent mPCNL demonstrated that CCI was more accurate than the CDC in illustrating the overall burden of postoperative complications and more precise prediction of length of hospital stay (LOS) [14]. Controversial results were also showed in the early literature. Triemstra et al. study also supported that comparing CCI to CDC for complication burden assessment, the former resulted in more work and no clinically significant benefit [16]. Similar to controversial results, another study was assessed CCI score and length of hospital stay after endourological surgeries and CCI was not provide more accurate prediction of LOS than CDC [15]. The authors commented that this is a results of low overall complication rates and CDC-dependent formulation. FGSI was identified to predict mortality in FG [12, 18]. The FGSI can predict mortality with a 75% likelihood and survival with a 78% probability for patients suffering from Fournier’s gangrene [18, 19]. The studies comment this score is useful however, controversial though were in the literature due to underscores prognosis [10]. To enhance FGSI, Yılmazlar et al. study demonstrated that incorporating age and disease dissemination to FGSI could predict more accurately mortality (AUC:0.947, 0.873–0.994) [10]. Additional another study which compared FGSI, Uludag FGSI [UFGSI], age-adjusted Charlson Comorbidity Index [ACCI] and surgical Apgar Score [sAPGAR] showed that older patients with comorbidities as well as a need for mechanical ventilation and blood transfusion are at higher risk of mortality [20]. In our study, significantly higher CCI scores were observed in non-survivor group. Additionally, age and CCI were also independent factors for the mortality. This supports the hypothesis that patients who did not survive exhibited significantly higher CCI scores, supporting the concept that mortality in FG is strongly associated with the cumulative burden of complications. Our analysis identified a CCI score greater than 36.60 as a robust threshold for predicting mortality with a high specificity 91.3%. This provides clinicians with a concrete data point for risk stratification during the postoperative period. The significantly higher CCI scores in our non-survivor cohort suggest that these patients have an increased cumulative complication burden (not just the most severe complication). This finding is consistent with previous studies in urologic surgery (e.g., radical cystectomy, PCNL) demonstrating the effectiveness of the CCI in assessing the overall burden of postoperative morbidity [14, 17]. Furthermore, the relevance of a high CCI in identifying patients who are on a downward path is highlighted by the considerable link it has with the occurrence of sepsis and serious complications in our non-survivor group.

Biochemical and hematological parameters also assessed to predict to mortality [21]. Early study Erol et al. showed that white blood count (WBC) and magnesium levels were different in survivors and non-survivors at the admissions (*p* = 0.008, and *p* = 0.018, respectively) [21]. Another study conducted by Demirci et al. showed that there was no significant different between survivor or non-survivor in terms of WBC and neutrophile and lymphocytes ration. However, that study also showed that there was significant change in hematological parameters after first debridement [22]. Another novel study also showed that c-reactive protein levels were similar between survivor and non-survivor groups (*p* = 0.354). However, this study also demonstrated that CRP-lymphocyte ratio were higher in non-survivor group (*p* = 0.008) [23]. In our study CRP levels were higher in survivor groups. This paradoxical pattern may result from a delayed presentation and swift decline in non-survivors, causing hemodynamic failure prior to the establishment of a complete inflammatory response, with vigorous initial fluid resuscitation or early organ dysfunction obscuring CRP increase.

The findings in the literature also focus on the improvement of the CCI with cumulative accumulation rather than only the CCI score. Huang et al. also found that the cumulative CCI and upgrading were more accurate predictors for postoperative stay than the CDC [17]. Similar to Huang et al. study, Kowalewski et al. conducted a study involving 682 patients who underwent radical cystectomy, prostatectomy, and partial nephrectomy to confirm the CCI benefits over CDC [24]. Their findings indicated that CCI upgraded the Clavien classification for 2.4–32.4% of patients and CCI was more accurate in predicting LOS. Additionally, Vetterlein et al. showed that the CCI increased with every CDC grade overall 20% of patients were up-graded to major complication level after calculating cumulative CCI [25]. Supporting these results, our study indicates that CCI upgrading significantly higher in mortality group. while CCI was significantly higher in mortality group, CDC score was also higher in mortality group. We believe the primary reason for this is that the CCI is a CDC-dependent tool. However, the post-cumulative upgrading is still valuable tool to determine morbidity.

The use of CCI in FG may facilitate the early identification of high-risk patients and the development of appropriate treatment plans. We believe that this is because every complication and treatment—or the need for more than one intervention—in diseases such as FG, especially in fragile patients, has a cumulative impact on mortality. Our study highlights that patients with a high CCI score experience sepsis and major complications more frequently, emphasizing the need for a multidisciplinary approach in these cases. The integration of CCI into routine clinical practice could enable the effective utilization of resources and improve postoperative care processes.

This study has several limitations. Its retrospective, single-center design may introduce selection bias and limit the generalizability of our findings. The modest sample size, while typical for a rare disease like Fournier’s gangrene, reduces the statistical power of our analysis. We were unable to include potential confounding factors, like the CDC (a factor dependent on CCI data). However, our study showed predictive effect of CCI in patients with Fournier gangrene for the first time. Future research should focus on prospective, multicenter studies to validate our proposed CCI cutoff value and confirm the distinct prognostic roles of FGSI and CCI in a larger, more diverse cohort.

## Conclusion

This study underscores the critical role of the Comprehensive Complication Index (CCI) in predicting outcomes for Fournier’s gangrene patients. Our results show that the CCI score effectively assesses postoperative morbidity and mortality, offering greater accuracy than traditional classification systems like the Clavien-Dindo Classification (CDC). A CCI score above 36.60 strongly predicts mortality, highlighting its value in risk stratification. Additionally, age and comorbidity burden emerged as independent mortality predictors. The higher incidence of sepsis and complications in patients with elevated CCI scores calls for early recognition and management. Integrating the CCI into clinical practice could enhance decision-making, improve postoperative care, and optimize resource allocation. Future multicenter studies are needed to confirm these findings and refine CCI’s clinical application. 

## Data Availability

We can share our data when requested.
